# Dose-Volume Response Relationship for Brain Metastases Treated with Frameless Single-Fraction Linear Accelerator-Based Stereotactic Radiosurgery

**DOI:** 10.7759/cureus.587

**Published:** 2016-04-27

**Authors:** Mark Amsbaugh, Jianmin Pan, Mehran B Yusuf, Anthony Dragun, Neal Dunlap, Timothy Guan, Warren Boling, Shesh Rai, Shiao Woo

**Affiliations:** 1 Radiation Oncology, University of Louisville; 2 JG Brown Cancer Center, University of Louisville; 3 Neurosurgery, University of Louisville; 4 Bioinformatics and Biostatistics, University of Louisville

**Keywords:** frameless stereotactic radiosurgery, frameless radiosurgery, brain metastasis, brain mets, single fraction, dose-volume response, dose volume

## Abstract

Background: Our aim was to identify a dose-volume response relationship for brain metastases treated with frameless stereotactic radiosurgery (SRS).

Methods: We reviewed patients who underwent frameless single-fraction linear accelerator SRS for brain metastases between 2007 and 2013 from an institutional database. Proportional hazards modeling was used to identify predictors of outcome. A ratio of maximum lesion dose per mm-diameter (Gy/mm) was constructed to establish a dose-volume relationship.

Results: There were 316 metastases evaluated in 121 patients (2 - 33 mm in the largest diameter). The median peripheral dose was 18.0 Gy (range: 10.0 – 24.0 Gy). Local control was 84.8% for all lesions and was affected by location, peripheral dose, maximum dose, and lesion size (p values < 0.050). A dose-volume response relationship was constructed using the maximum dose and lesion size. A unit increase in Gy/mm was associated with decreased local failure (p = 0.005). Local control of 80%, 85%, and 90% corresponded to maximum doses per millimeter of 1.67 Gy/mm, 2.86 Gy/mm, and 4.4 Gy/mm, respectively. Toxicity was uncommon and only 1.0% of lesions developed radionecrosis requiring surgery.

Conclusions: For brain metastases less than 3 cm, a dose-volume response relationship exists between maximum radiosurgical dose and lesion size, which is predictive of local control.

## Introduction

An estimated 20-40% of all cancer patients will develop metastatic disease to the brain [1]. The incidence of brain metastases will likely continue to rise as improvements in systemic therapy increase patient survival, and sensitive imaging techniques are more widely adopted. Despite recent advances in treatment, the prognosis of patients with brain metastases remains poor [2]. Independent of being a negative predictor of survival, brain metastases in eloquent areas and proximal to critical structures can also threaten the neurocognitive quality of life by compromising sensorimotor function or language. Given that individual patients demonstrate a wide range of survival, identifying parameters for maximal local control (LC) of brain metastases is critical to optimizing both survival outcomes and quality of life [3]. It is likely that these factors will become increasingly important as control of systemic disease improves.

The goal of therapy for brain metastases is the long-term control of the disease with minimal toxicity. Stereotactic radiosurgery (SRS) has been established by multiple randomized trials as an effective modality for treating limited metastases [4-7]. SRS used alone has been shown to be effective for the treatment of select patients and is now commonly used in a wide range of patients [4]. The Choosing Wisely® campaign now recommends not routinely adding WBRT to radiosurgery for patients with limited brain metastases [8]. SRS control of a metastasis has been demonstrated to increase with dose [9-12]; however, both larger doses and volumes may result in higher toxicity [13-15].

Normal tissue toxicity of the brain after therapeutic radiation has been extensively investigated, and a relationship between dose and the development of radiation necrosis has been demonstrated [13-14]. Refining and better describing this relationship have been an area of active investigation. The RTOG 90-05 study sought to define dose parameters for brain lesions treated with SRS based upon tumor diameter, and these dosing parameters have subsequently been widely adopted by subsequent investigations, including RTOG 95-08 [16]. These studies were not designed to establish optimal SRS dosing based on lesion size, but rather sought to identify the maximum tolerable dose of single fraction radiosurgery based on acceptable rates of CNS toxicity. The SRS dose selection for a given treatment volume must balance a dose that is high enough to control the lesion without increasing the risk of toxicity. While properly selected dose parameters can result in acceptable rates of neurotoxicity, there remains a debate regarding the optimal SRS dose selection, which balances lesion control while minimizing toxicity [9].

The optimal dose for brain metastases treated with upfront SRS is unknown. In this study, we examined frameless linear accelerator SRS cases from a single institution.  We sought to identify trends that exist in patients treated with an empiric dose selection in order to identify a dose-volume response relationship for tumor control.

## Materials and methods

### Patients

As part of this University of Louisville Institutional Review Board-approved study, we examined the records of all patients who underwent frameless linear accelerated-based radiosurgery at a single institution treated by seven radiation oncologists between 2007 and 2013. Of the 136 patients who received SRS for one or more brain metastasis, six patients were excluded because their tumors were surgically removed prior to treatment and nine patients were excluded because they underwent fractionated radiosurgery (usually due to large lesion size). For the remaining 121 patients, 316 individual metastases were treated. Patients were generally followed with magnetic resonance (MR) imaging of the brain every three months for the first year after radiosurgery. After the first year, neuroimaging was performed every four to six months. 

### Radiosurgery

**Figure 1:**
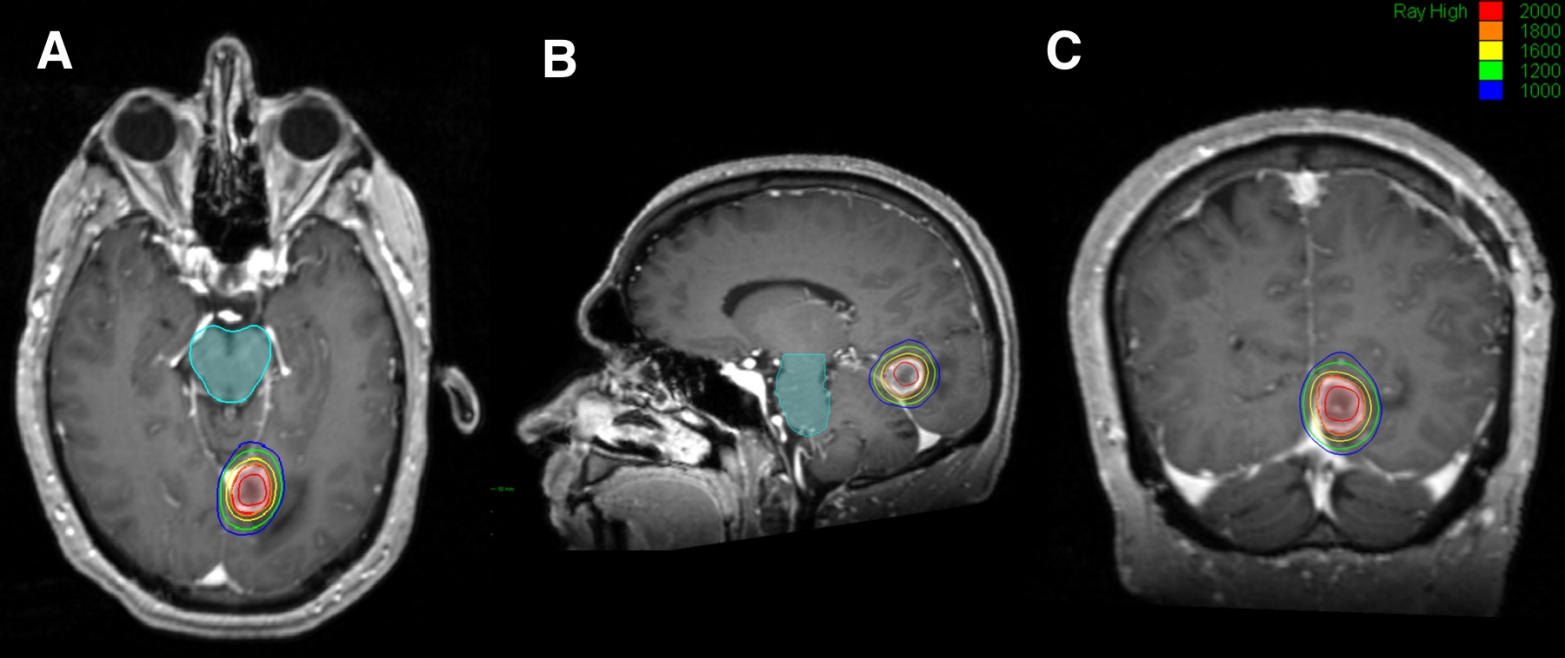
Example CyberKnife Treatment Plan

Three hundred frameless single fraction radiosurgical treatments (94.9%) were delivered using the Varian Trilogy linear accelerator (Varian, Palo Alto, CA) and 16 (5.1%) were delivered using the CyberKnife linear accelerator (Accuray, Sunnyvale, CA). Patients were simulated supine with a thermoplastic mask placed for immobilization. An MR image of the brain with 1 mm axial slices was fused to the planning CT. Planning target volume was defined as the contrast-enhancing tumor, incorporating both subclinical disease and uncertainties in plan delivery. No additional margin was added for treatment planning to minimize dose to adjacent peripheral normal brain tissue and the subsequent risk of toxicity associated with larger target volumes [4]. Plans were constructed using three to five (median: 4) non-coplanar arcs (293 metastases), a volume modulated arc therapy technique (seven metastases) or a multiple-node pencil beam technique (16 metastases). An example plan constructed for delivery using the CyberKnife linear accelerator has been included for reference (Figure 1). The dose was prescribed to the tumor margin. Prescribed dose and isodose line were selected at the discretion of the treating radiation oncologist empirically based on lesion size and did not exceed safe levels suggested by RTOG 90-05 [15]. Intrafraction motion management was performed using an infrared mouthpiece or orthogonal x-rays (CyberKnife skull tracking). Patients were discharged the same day and prophylactic steroids were not routinely used.

### Statistical analysis

Patient characteristics including age, sex, performance status, extracranial disease status, primary disease site, histology, and the number of metastases at presentation were collected. Tumor and treatment-related factors, including maximum lesion diameter, brain location, prior brain radiotherapy, treating radiation oncologist, the technique used, peripheral dose, isodose, and maximum dose, were collected.

The primary outcome variable, local failure (LF), was defined as retreatment of the lesion with radiotherapy, surgical resection, or 25% increase in the size of the largest dimension of a treated metastasis on two serial MR images. Overall survival (OS) was defined as the time from first radiosurgical procedure until death. Neurologic death was defined as death with progressive intracranial disease. Toxicity was coded according to the Common Terminology Criteria for Adverse Events, version 4.03. Advanced imaging techniques, including MR spectroscopy, MR perfusion, and diffusion-weighted sequences, were used to aid in differentiating radiation necrosis from possible disease progression. All cases of unclear treatment effect or tumor progression were presented at an institutional multidisciplinary tumor board with neurosurgeons, neuroradiologists, neurooncologists, and radiation oncologists for a consensus of opinion.

The secondary endpoint, OS, was analyzed by the Kaplan-Meier technique and the log-rank test was used to estimate the OS time. The Cox proportional hazard model was used to analyze factors predictive for OS, LF, or toxicity and provide hazard ratio and associated Wald 95% confidence interval for each model. Analyses were all two-sided. Dose-volume-response curves with 95% confidence limits were constructed using maximum target dose divided by lesion diameter (Gy/mm) plotted against predicted control rate by fitting the univariable logistic regression model using the maximum likelihood method. Volumetric tumor size was not available for all lesions. Maximal axial dimension was used as a surrogate for tumor volume based on the findings of multiple studies supporting a strong correlation between greatest axial dimension and tumor volume [17-19]. Values of maximum dose per lesion diameter were considered both as a continuous variable and a categorical variable, and cut-points were established for lesion control and toxicity using equal increments. Statistical analyses were performed using methods described by Mathews and Farewell [20], Rosner [21], Yuan and Rai [22], and Walker and Shostak [23] using SAS (The SAS Institute, Cary NC).

## Results

Three hundred and sixteen metastases in 121 patients were treated using single-fraction SRS. Patient characteristics are shown in Table 1 and tumor characteristics are shown in Table 2. Most patients treated with radiosurgery had non-small cell lung cancer (53 patients), melanoma (28 patients), or breast cancer (15 patients). The majority of patients presented with one (53 patients), two (30 patients), or three (22 patients) metastatic lesions in the brain but may have subsequently developed additional metastatic lesions.

**Table 1:** Patient Characteristics

Characteristic	Number (%)
Age	
< 40 years	4 (3.3)
40 - 50 years	16 (13.2)
51 - 60 years	35 (28.9)
60 - 70 years	42 (34.7)
> 71 years	24 (19.8)
Karnosfsky performance status	
90 - 100%	54 (44.6)
70 - 80%	61 (50.4)
< 70%	6 (5.0)
Primary histology	
Non-small cell lung	53 (43.8)
Melanoma	28 (23.1)
Breast	15 (12.4)
Small-cell lung	13 (10.7)
Other	12 (9.9)
Extracranial disease	
Controlled	102 (84.2)
Uncontrolled	19 (15.7)

**Table 2:** Radiosurgery Characteristics WBRT: whole brain radiotherapy, SRS: stereotactic radiosurgery

Characteristic	Number(%)
Lesion size	
< 5 mm	50 (15.8)
5 - 10 mm	145 (45.9)
11 - 20 mm	88 (27.9)
21 - 30 mm	32 (10.1)
> 30 mm	1 (< 1.0)
Brain location	
Frontal lobe	80 (25.3)
Temporal lobe	45 (14.2)
Parietal lobe	68 (21.5)
Occipital lobe	41 (12.9)
Cerebellum	63 (19.9)
Midbrain	16 (5.1)
Brainstem	3 (1.0)
Peripheral dose	
< 16 Gy	36 (11.4)
16-20 Gy	247 (78.2)
> 20 Gy	33 (10.4)
Prescription isodose	
< 70%	13 (4.1)
71 - 80 %	254 (80.4)
81 - 90 %	41 (13.0)
> 90 %	8 (2.5)
Maximum dose per size	
< 2.5 Gy/mm	153 (48.4)
2.5 - 5 Gy/mm	117 (37.0)
5 - 7.5 Gy/mm	27 (8.5)
7.5 - 10 Gy/mm	8 (2.5)
> 10 Gy/mm	11 (3.5)
Pre-SRS steroids	
Yes	119 (37.7)
No	197 (62.3)

Treated brain metastases ranged from 2 – 33 mm (median: 8 mm) in largest diameter. Most lesions were treated with SRS alone (92.7%); however, some were treated with WBRT with an SRS boost (7.3%). Two hundred and fifty-nine lesions were treatment-naive at the time of SRS and 57 were treated in the salvage setting (having received some previous radiotherapy). The dose was prescribed to the tumor margin (median peripheral dose: 18 Gy, range: 10-24 Gy). The prescription isodose line was chosen individually for each lesion (median prescription isodose: 80%, range: 60-96%). Maximum dose (highest dose to a point) was calculated and used for plan evaluation purposes (median maximum dose: 22.5 Gy, range: 11.1 – 33.3 Gy). For purposes of this analysis, a maximum target dose per size parameter was constructed. Lesions were treated with 0.53 – 16.67 Gy/mm (median: 2.66 Gy/mm).

**Figure 2:**
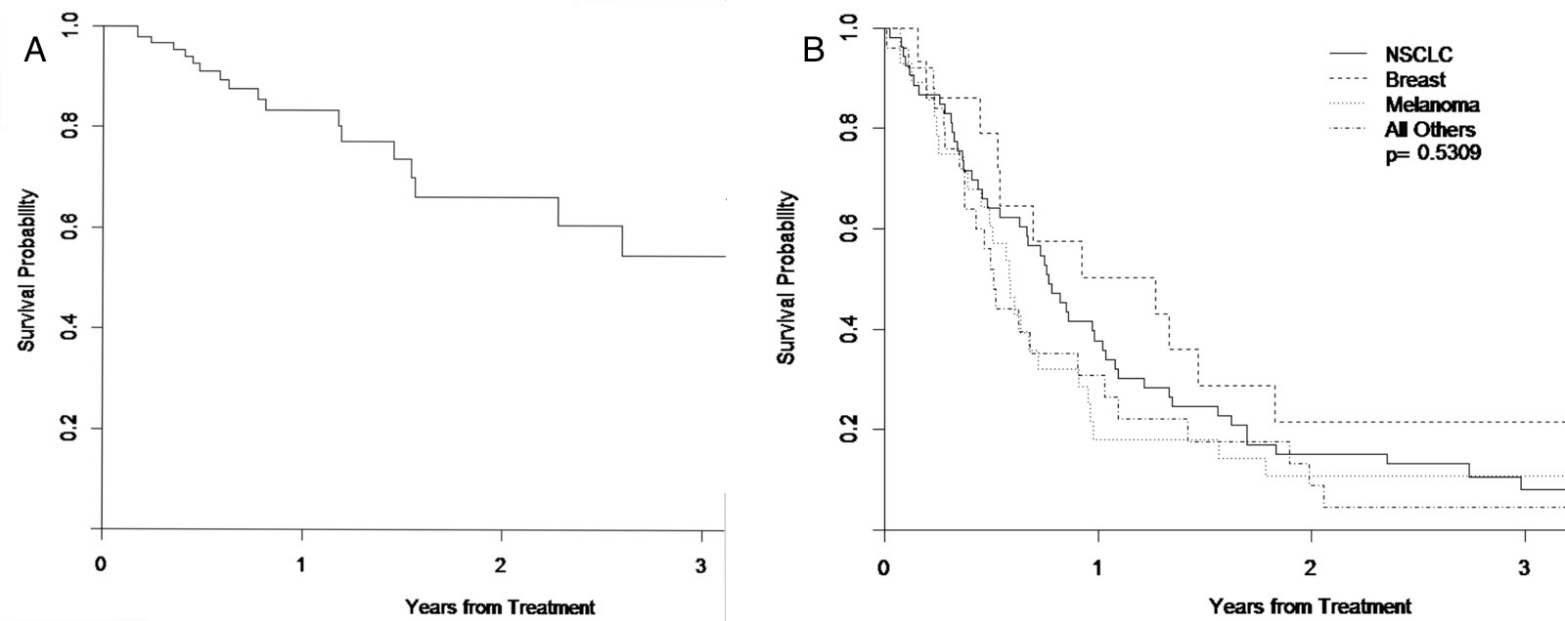
Estimated Event-free Survival and Overall Survival for All Patients Figure 2A shows Kaplan-Meier analysis of event-free survival Figure 2B shows overall survival of all patients by primary tumor type

**Figure 3:**
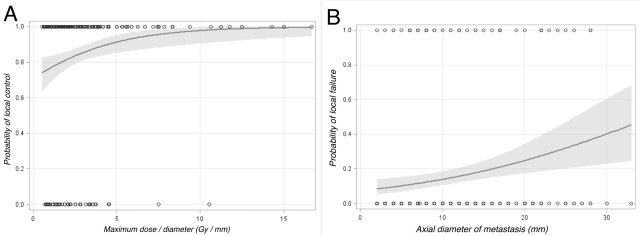
Predictors of Local Control for All Treated Brain Metastases Figure 3A shows predicted probability of local control by maximum dose per size considered as a continuous variable Figure 3B shows local failure probability by axial diameter of metastasis.

Forty-eight lesions met the criteria for LF. Actuarial LC at 6 months and 12 months were 91.0% (95% CI 81.4-95.9%) and 83.4% (95% CI 70.2-90.8), respectively (Figure 2). Univariate logistic regression demonstrated that location (p = 0.041), maximum dose (p = 0.025), lesion size (p = 0.001), and maximum dose per size (p = 0.005 categorical, p = 0.008 continuous) significantly correlated with LC. Peripheral dose did not meet criteria for statistical significance (p = 0.070). Local failure was more common with lower maximum or peripheral dose, larger lesions (Figure 3), or lower maximum dose per size. On multivariate analysis, location in the brainstem (HR 13.072 95% CI 1.141-149.731) and maximum dose per size (HR 0.740 95% CI 0.600-0.913) were significantly correlated with LC. No significant differences were seen based on primary histology (p = 0.138), treating oncologist (p = 0.777), brain lobe (p = 0.221), or treatment setting (upfront or salvage) (p = 0.607).

Maximum dose per size was selected as a measure because it incorporates peripheral dose, prescription isodose line, and lesion size into one variable. Predicted probability for LC for a given Gy/mm is shown in Figure 3 and Table 3. This parameter of maximum dose per size was found to correlate with local failure for patients treated for a de novo lesion (HR 0.707 95% CI 0.557 – 0.897) and for patients treated in the salvage setting (HR 0.764 95% CI 0.422 – 1.386) but did not reach statistical significance in the salvage setting likely secondary to only 57 lesions being treated. Likewise, this parameter was correlated regardless of if the patient had previously received WBRT (HR 0.708 95% CI 0.467-1.072) or not (HR 0.751 95% CI 0.593-0.951).

**Table 3:** Predicted Probability of Local Control for a Given Maximum Dose Per Size

Dose (Gy/mm)	Probability (95% CI)
0.5	74% (63 - 83%)
1.0	77% (68 - 83%)
2.0	82% (76 - 86%)
3.0	86% (81 - 89%)
4.0	89% (84 - 93%)
5.0	91% (85 - 95%)
6.0	93% (86 - 97%)
7.0	95% (88 - 98%)

Examining the dose-volume response relationship (Figure 3) a cut-point was determined to be 2.5 Gy/mm, using equal increments corresponding to a predicted LC of 83.6% (95% CI 80.0 – 87.7%). One hundred and seventy-seven lesions were treated with a maximum dose equal to or greater than 2.5 Gy/mm with a crude LC of 89.2% compared to 63.3% for lesions treated with a lower dose.

At the time of analysis, all but eight patients were deceased. Actuarial median OS was 8.1 months (95% CI 6.4 – 10.3 months). Figure 2 shows OS for all patients. Thirty-four of 121 patients (28.1%) experienced a neurologic death.

Overall, 6.9% of tumor sites developed Grade 3 or higher toxicity. No patient experienced grade 5 toxicity. The most common toxicity was necrosis (11 tumor sites Grade 3, two tumor sites Grade 4). Grade 3 or 4 necrosis developed in 4.1% of all tumor sites. Nine tumor sites were determined to be the cause of post-SRS seizures. Univariate logistic regression demonstrated that only max-dose/diameter was significantly associated with toxicity. Smaller tumors had a lower rate of developing a Grade 3 or higher toxicity than larger tumors. Tumors measuring from 0 - 20 mm had a toxicity rate of 3.9% compared to a rate of 18.75% for tumors 21 – 30 mm. Only 1.9% of tumor sites treated with a dose equal to or greater than 2.5 Gy/mm developed Grade 3 or higher toxicity.

## Discussion

With improvements in systemic therapy, LC of brain metastases will continue to be an increasingly important component of care for patients with metastatic cancer. Previously, radiosurgery prescription doses for brain metastases have been based on the RTOG 90-05 dose escalation study or institutional preference [15]. These dosing strategies take into account the maximal safe dose but do not consider lesion control. Our results demonstrate a dose-volume response for the first time for small brain metastases treated with single fraction SRS, identify a new dose parameter predictive of tumor control, and provide a method for predicting local tumor response based on that parameter (Table 3).

In our data, size and LC were correlated, with larger lesions having higher rates of failure than smaller lesions.  Previous studies have supported this correlation [10, 11]. Chang et al. examined 153 brain metastases in 135 patients treated with linear-accelerator based radiosurgery and found size to be the only significantly correlated variable with tumor control on multivariate Cox regression analysis (size greater than 1cm, HR 3.53 95% CI 1.53 – 8.13). In their report, lesions larger than 1 cm had a estimated LC at 1-year of 56% compared to 86% for lesions smaller than 1 cm [10]. Chao and colleagues later confirmed these results and suggested that despite the fact that increasing lesion size was correlated with worse LC, brain metastases up to 2 cm may still have an excellent LC rate [11]. Lesions 2 cm or less had 1-year LC of 91% compared to 62% of lesions larger than 2 cm [11].

Increasing lesion size also leads to increased risk of toxicity, likely as a result of increasing dose.  The volume of brain receiving at least 12 Gy (V12Gy) has been demonstrated to be at risk for radionecrosis [13, 14].  Larger target sizes exponentially increase the volume of brain at risk [24], and translate into increased toxicity [15]. Because of this, prescribed dose for large lesions has been lower than for smaller lesions, contributing to a lower LC rate. While our toxicity rate was higher with larger targets, it remained acceptable by strictly adhering to the constraints established by the RTOG 90-05 study [15]. Given the higher toxicity rates seen with lesions over 3 cm, most lesions of this size are treated with fractionated stereotactic radiosurgery (FSRT) at our institution. It is important to note lesion size was small in our population sample with a median diameter of 8 mm and with only 2 tumors greater than 3 cm, our results may not be applicable to lesions exceeding this size.

Delivered dose for SRS is a result of marginal dose and prescription isodose. We observed a strong correlation between maximum target dose and marginal dose. Previous studies have attempted to show a correlation between margin dose and maximum dose with tumor control [9-12, 25-29]. Vogelbaum et al. evaluated 375 metastases in 202 patients in a large study examining the effect of SRS dose on the LC of brain metastases [28]. They found a significantly lower risk of local failure in patients who received a marginal dose of at least 24 Gy than patients who received 15 or 18 Gy (HR 0.277, 95% CI 0.134-0.573), but no difference between those who received 15 or 18 Gy (p = 0.82). Maximum target dose has also been shown to be an important independent prognostic factor for brain metastasis control following SRS. Noel et al. examined 145 brain metastases in 92 patients and determined maximum tumor dose to be the prognostic factor for LC with the highest correlation [12].

In our study, maximum dose per size was significantly correlated with LC for lesions treated with SRS alone. However, this relationship was not demonstrated to be significant for lesions treated with WBRT in addition to SRS, likely given the much lower patient numbers in that group. In the previously referenced studies, a high percentage of patients received either prior whole brain radiotherapy or as part of the treatment course. However, in our study, only 7.3% of metastases were treated with WBRT with a SRS boost. Prior studies have demonstrated WBRT before or at time of SRS improved local tumor control [4, 9, 27]. De Azevedo et al. examined 305 brain metastases in 141 patients, with 56% of lesions having had no previous exposure to WBRT [9]. Although WBRT was correlated with LC on univariate analysis, dose was the only significant factor on multivariate analysis, suggesting that for patients who receive WBRT, the additional dose is the primary driver of improved LC [9].

Delivered dose (including prescription dose and isodose prescription) and target size are intricately intertwined with both tumor response and toxicity to the surrounding brain. While both have previously been shown to correlate with tumor control, the concept of maximum dose per lesion diameter incorporates size, dose, and prescription isodose into one parameter. Our model can be used to estimate LC of a brain metastasis after single fraction radiosurgery given the maximum delivered dose and lesion size.

Our model suggests a sharp fall-off in local tumor control occurs below a cut-point of 2.5 Gy/mm. Whether higher values of this parameter can be accomplished safely depends on both treatment planning and lesion size. While small tumors can readily be treated to high Gy/mm values, larger tumors are more often limited by the maximal tolerated dose determined by safety constraints. Maximum doses of over 35 Gy are difficult to achieve using linear accelerator based SRS while respecting constraints established by RTOG 90-05. This corresponds to a lesion size of 14 mm using our proposed cut-point. It is important to consider, that according to our model, higher maximum dose per lesion size are predictive of local control even if that value is below the 2.5Gy/mm cut-point. Our data suggests that high maximal doses should be used while respecting previously established toxicity guidelines.

Survival is complex in patients with brain metastases, and primarily determined by factors such as performance status, age, number of metastases, primary diagnosis and presence of extracranial metastases are more important predictors of survival than intracranial tumor control [2]. Our rate of neurologic death of 28.1% was consistent with that of other recent studies of SRS for brain metastases [30]. Local control of brain metastases may become more clinically relevant as improving systemic therapies continue to prolong survival with metastatic disease, potentially increasing intracranial control’s influence on survival.

Limitations exist with these data. This was a retrospective analysis of data from a single institution and is therefore vulnerable to the inherent biases affecting all similar studies. Lesions included in this study were small, and the results may not be applicable to brain metastases over 3 cm. The dose-volume response relationship is complicated for brain metastases. Factors contributing to local tumor control are difficult to determine given the heterogenous doses and treatment techniques used to treat brain metastases with SRS. Prospective dose escalation trials are needed to evaluate maximum dose per lesion size to confirm the clinical significance of this parameter.

## Conclusions

Our study demonstrates that in a large series of brain metastases treated with linear accelerator based SRS, both maximum dose and lesion size are important predictors of LC. Furthermore, a dose-volume response ratio exists and is predictive of local treatment outcome for small lesions. These factors can be combined into a dose parameter of Gy/mm that can be used to independently predict LC during plan evaluation for selected dosing, which should respect previously established constraints. 
